# Development and validation of assessments of adolescent health literacy: a Rasch measurement model approach

**DOI:** 10.1186/s12889-022-12924-4

**Published:** 2022-03-25

**Authors:** Sasha A. Fleary, Karen M. Freund, Claudio R. Nigg

**Affiliations:** 1 Current address: Department of Community Health and Social Sciences, City University of New York Graduate School of Public Health and Health Policy, 55 W 125th St, New York, NY 10027 USA; 2 Eliot-Pearson Department of Child Study and Human Development, Tufts University, 105 College Ave, Medford, MA 02155 USA; 3 Department of Medicine, Tufts University School of Medicine, 800 Washington St, Boston, MA 02111 USA; 4 Department of Health Science, Institute of Sports Science, University of Bern, Bremgartenstrasse, 145 3012 Bern, Switzerland

**Keywords:** Adolescents, Health literacy, Measurement, Health communication

## Abstract

**Background:**

Health literacy (HL) is implicated in improved health decision-making and health promotion, and reduced racial, ethnic, and socioeconomic health disparities. Three major areas of HL include functional, interactive, and critical HL. HL skills develop throughout the lifespan as individuals’ psychosocial and cognitive capacities develop and as they accumulate experiences with navigating health systems. Though adolescence is marked by increased involvement in health decision-making, most HL studies and measures of HL have focused on adults. Both the adult and adolescent HL literature are also limited by the paucity of validated test-based measures for assessing HL. The existing test-based validated HL measures for adolescents were originally designed for adults. However, adolescents are at an earlier phase of developing their HL skills (e.g., fewer experiences navigating the health system) compared to adults and measures originally designed for adults may assume prior knowledge that adolescents may lack therein underestimating adolescents’ HL. This study developed and validated test-based assessments of adolescents’ functional, interactive, and critical HL.

**Methods:**

Items were generated in an iterative process: focus groups with adolescents informed item content, cognitive interviews with adolescents and expert consultation established content and face validity of the initial items, and items were revised or removed where indicated. High school students (*n* = 355) completed a measurement battery including the revised HL items. The items were evaluated and validated using Rasch measurement models.

**Results:**

The final 6-item functional, 10-item interactive, and 7-item critical HL assessments and their composite (23 items) fit their respective Rasch models. Item-level invariance was established for gender (male vs. female), age (12–15-year-olds vs. 16–18-year-olds), and ethnicity in all assessments. The assessments had good convergent validity with an established measure of functional HL and scores on the assessments were positively related to reading instructions before taking medicine and questioning the truthfulness of health information found online.

**Conclusions:**

These assessments are the first test-based measures of adolescents’ interactive and critical HL, the first test-based measure of functional HL designed for adolescents, and the first composite test-based assessment of all three major areas of HL. These assessments should be used to inform strategies for improving adolescents’ HL, decision-making, and behaviors.

**Supplementary Information:**

The online version contains supplementary material available at 10.1186/s12889-022-12924-4.

## Background

In adults, health literacy (HL) is implicated in a broad array of health behaviors, healthcare utilization, and outcomes including the use of prevention and emergency services, medical adherence, and interactions with providers [1–5]. Further, HL has been identified as a key mediator of racial, ethnic, and socioeconomic health disparities in multiple contexts [6, 7]. As such, HL has emerged as an important target for improving health decision-making and health promotion, and reducing racial, ethnic, and socioeconomic health disparities [8]. In Sørenson and colleagues’ [8] comprehensive review of 17 definitions and 12 conceptual frameworks of individual-level HL, they summarized the concept into two dimensions: 1) core qualities of HL and 2) scope and reach of its applied use. According to Sørenson and colleagues [8], Nutbeam’s [9] population health-oriented definition of HL falls within the core qualities of HL. Nutbeam’s [9] definition includes three major areas: functional HL (FHL; reading, writing, numeracy skills related to health information), interactive HL (IHL; use of health knowledge to communicate and interact with others and environment), and critical HL (CHL; advocacy for self and others through sociopolitical action).

Sørenson and colleagues [8] argue that HL skills develop throughout the lifespan as individuals’ psychosocial and cognitive capacities develop and as they accumulate experiences with navigating health systems. Therefore, though most studies focus on adulthood, HL skills development begins earlier in the lifespan. Adolescence, in particular, is marked by increased cognitive capacity for and involvement in health decision-making and is therefore a salient period for developing and using HL skills [10]. Yet, adolescents’ HL is understudied [11]. Several studies have established the relationship between adolescent HL and health behaviors [12, 13]. However, in their review of the literature, Fleary and colleagues [11] found that most studies exploring the relationship between adolescents’ HL and health behaviors predominantly assessed FHL. In their qualitative research, Fleary and Joseph [14, 15] found that the HL skills adolescents use in their health decision-making encompass Sørenson and colleague’s [8] HL definition and Nutbeam’s [9] FHL, IHL, and CHL paradigm. For example, adolescents described being able to ask questions at their doctor’s appointments (IHL) and critically analyze health information provided to them (CHL) as examples of good health decision-making skills. Therefore, it is important that these HL skills be considered in determining the role of HL in adolescents’ health decision-making and behaviors and how best to intervene on HL to improve health outcomes for adolescents.

Both the adult and adolescent HL literature are limited by the paucity of validated test-based measures for assessing the core qualities of HL outlined by Nutbeam [9]. Test-based measures assess skills and knowledge rather than perceptions. Despite the multiple definitions of HL over the years [8], most measures of HL focus primarily on FHL. For example, the Rapid Estimate of Adult Literacy in Medicine-Short Form (REALM-Short Form) is a 7-item word recognition test used in clinical settings [16]. The Short-Test of Functional HL in Adults is a 40-item scale of reading and numeracy [17]. The Newest Vital Sign (NVS) [18] is a 6-item measure of reading and numeracy. All of these measures were initially validated on adults. However, there is now a validated 10-item REALM-Teen short form [19] and a validation study suggesting that the NVS is valid for assessing FHL in adolescents [20]. In the adolescent HL literature, there is a growing number of perceptions-based HL measures that include IHL and CHL [21–25]. However, scales that measure perceived HL via self-report tools may not align with actual competency and hinder actions one takes to become competent [26, 27]. Further, using measures of perceived competencies to develop interventions may lead to misaligned programming, resulting in ineffective interventions and wasted resources. Hence, the need for test-based HL measures.

The existing test-based validated HL measures for adolescents were originally designed for adults. However, adolescents are at an earlier phase of developing their HL skills (e.g., fewer experiences with navigating the health system, cognitive and psychosocial development immature compared to adults) compared to adults and, though validated, measures originally designed for adults may assume prior knowledge that adolescents may lack. Hence, the need to assess developmentally appropriate HL skills. For example, the NVS asks questions based on a nutrition label and prior exposure to nutrition labels may make it easier for the patient to answer the questions. However, younger adolescents in particular may score poorly on this because they lack prior experience rather than due to lack of literacy and numeracy skills for health decision-making. Therefore, it is important that HL measures for adolescents are especially designed to account for their developmental characteristics and experiences (e.g., daily preventive health behaviors).

This study developed and validated test-based assessments of adolescents’ FHL, IHL, and CHL using the Rasch measurement model. Rasch, a probabilistic model, involves testing data fit against a measurement model rather than a specific sample as is done in classical test theory [28]. Analyses entail calculating the probability of a particular person responding in a particular manner to a particular item. Persons with higher ability have higher probabilities of endorsing items whereas items with higher difficulties have lower probabilities of being endorsed. Item difficulty is estimated independent of the sample and person ability is estimated independent of the items in the measure [29]. Cutoff scores distinguishing levels of ability can be determined. Hence, Rasch is appropriate for developing assessments of adolescents’ HL skills. We hypothesized that the final assessments would have good convergent validity with a previously validated measure of FHL. We also compared adolescents’ HL test scores with their self-reported HL-related behaviors to establish criterion validity.

## Methods

### Study design

A multi-phase mixed methods design was utilized to develop and validate Assessments of Adolescent Health Literacy (AAHL). As a secondary step, a composite of finalized FHL, IHL, and CHL assessments was modeled. This study was approved by the Tufts University Social Behavioral and Educational Institutional Review Board. Parent permission and adolescent assent were obtained for adolescents’ participation at each phase of data collection.

### Measures

#### Demographics

Participants indicated their age in years, gender (male, female, transgender, non-binary, other, and prefer not to answer), ethnicity (Hispanic, Latino/a or of Spanish origin), and race (Black or African American, Asian, Native America or Alaskan Native, Native Hawaiian or Other Pacific Islander, White, and Other). Participants selecting multiple races were categorized as multiracial.

#### Newest vital sign

The NVS [18], a 6-item measure of FHL with good internal consistency (Cronbach α = 0.76), was used to evaluate convergent validity with the AAHL. Participants responded to six reading and numeracy questions about the information on an ice cream label. Responses were scored, summed, and categorized into high likelihood of limited literacy (0–1 correct), possibility of limited literacy (2–3 correct), and adequate literacy (≥4 correct). Summed scores were used for calculating convergent validity. The NVS has good criterion validity with the Gray Silent Reading Test in adolescents [20].

#### HL behaviors

Participants indicated whether they engaged in two behaviors indicative of HL: questions the truthfulness of health information found online and reads instructions before taking medicine. These items were developed for this study based on focus groups where adolescents’ described how they knew they used HL skills [14]. These items are consistent with the scope and reach of applied use aspect of HL described by Sørenson and colleagues [8].

### AAHL development

Measurement development involved item bank development, quantitative data collection, and measurement evaluation.

#### Item Bank development

**Fig. 1:**
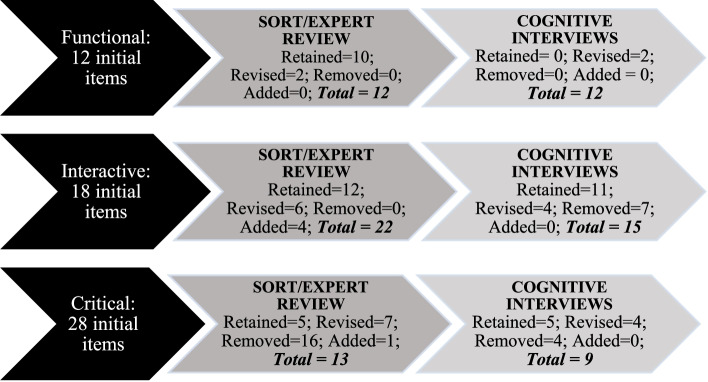
Illustration of iterative item bank revisions prior to large scale quantitative data collection

Six focus groups (~ 8 students/group) were conducted with 9th–12th grade high school students (Mean age = 16.49, Standard deviation [SD] = 1.35, 13–19-years, 86.5% girls, 35% non-Hispanic Black, 35% Hispanic/Latinx, 92% free/reduced lunch eligible) to better understand adolescents’ definition, operationalization, and use of HL (full results reported elsewhere [14, 15]). Focus groups were conducted after school and moderated by trained research assistants. During focus groups, participants also provided qualitative responses to scenarios related to IHL and CHL. Scenarios were developmentally appropriate in that it reflected experiences adolescents would have with health care, disease prevention, and disease promotion. The first author’s experience as a pediatric psychologist in hospital and community settings helped inform the scenarios. Scenarios were around obesity and obesity prevention, dental health, vaccinations, and understanding written health information. For example, participants were given a scenario about a low income family being given instructions by a doctor regarding changing behaviors to address a child’s obesity and was asked what would make it difficult for the family to follow the doctor’s recommendations and what can the community do to help families in similar situations. These focus groups were the first step to ensuring that the resulting assessments had content validity (measure adequately represents all aspects of the construct) and was developmentally appropriate [30]. The responses to the scenarios included in the focus groups were content-analyzed and used to inform response options to similar scenarios in the item bank and to develop other scenarios that would be familiar to adolescents. For example, based on the obesity scenario outlined above, adolescents’ responses were used to generate response options for a similar obesity scenario and attention was paid to the information in the scenarios that the participants were most responsive to so that short scenarios with only relevant information were created for the measure. Similarly, responses to questions about how adolescents use health literacy were used to develop relatable scenarios (e.g., interacting with family members about health choices, making decisions about healthy eating). Initial items were written by the first author and revised after feedback from the second and third authors and doctoral-level research assistants. Revisions included rewording items and adding and deleting information from scenarios. Items were cross-checked with focus groups data for content and consistency with adolescents’ responses and response styles. To establish face validity and further establish content validity, four graduate-level research assistants who completed substantial reading on HL and were involved in multiple HL projects but uninvolved in the item bank development engaged in a sorting activity to put items in three categories based on Nutbeam’s [9] definitions of FHL, IHL, and CHL. Two physicians who worked with adolescents were also provided with Nutbeam’s [9] definitions and the items and asked to indicate which type of HL the items belong to (if any) and provide feedback on current items and suggestions for additional items. Items were removed or revised if they were not unanimously sorted into the three categories. Items were also revised, removed, or added based on suggestions and feedback from the physicians. Next, adolescents 12–17-years-old were recruited from after-school settings to participate in cognitive interviews. Adolescents (*n* = 17, Mean age = 15.88 years, SD = 1.69, 47% girls, 41% non-Hispanic Black, 53% Hispanic/Latinx, 94% free/reduced lunch eligible) participated in the cognitive interviews while they completed the item bank questions. This process further established content validity as it provided data on how adolescents interpreted the questions and their thought processes as they responded. Cognitive interviews results were used to improve (e.g., rewording questions, calibrating the difficulties of the items) or remove problematic items. Figure 1 shows the iterative changes from initial item bank development to the revised item bank used for quantitative data collection. For both focus groups and cognitive interviews, data collection was discontinued once saturation was reached.

### Quantitative data collection and measurement evaluation

To refine the assessments further, the items were administered to a larger sample and Rasch measurement models were used to identify items that best fit the latent constructs of FHL, IHL, and CHL. A convenience sample of adolescents (12–18-years-old) was recruited from a local high school via flyers and classroom announcements. Data collection was coordinated with the school’s head health teacher and students completed the survey during health class. Some of the health classes were required and introductory-level while others were electives for adolescents who were interested in health careers. All students present completed the surveys, however, only data from students with signed permission and assent forms were used for study purposes (*n* = 355). The survey was administered via Qualtrics on researcher-provided tablets. Students received a $15 gift card for their participation.

### Statistical analyses

Rasch measurement models were estimated in Winsteps [31] and all other analyses were conducted in SPSS 27 [32].

FHL and IHL items were analyzed using the Rasch dichotomous model. CHL items were analyzed using the Rasch Partial Credit Model as response options were indicative of different degrees of CHL. Key assumptions of Rasch include unidimensionality, local independence, and monotonicity of the latent trait. Unidimensionality, whether items assessed a shared latent construct, was evaluated by examining the principal components analysis of the residuals [33]. If the eigenvalue of the unexplained variance in the first contrast was < 2, then unidimensionality was assumed [34]. Local independence, whether item responses were statistically independent of each other, was assessed by examining the standardized residual correlations of the items. Items with correlations < 0.50 were considered independent [35]. Monotonicity of the latent trait, whether scores were monotonically non-decreasing across the latent trait, was confirmed by monotonically ascending test characteristic curves [36].

Joint maximum likelihood estimation was used to estimate person and item parameters. Fit statistics included infit and outfit mean-squares and their standardized equivalents for both parameters. Mean-squares 0.5–1.5 indicated good fit and those < 0.5 or 1.6–2.0 were considered unproductive for measurement but did not degrade the measure. Standardized statistics > 2 or < − 2 indicated significant misfit [37]. Outfit statistics are most sensitive to misalignment of person responses and item difficulties. Item quality was judged by first identifying items with outfit mean-squares > 1.5, for these items, outfit standardized statistics were evaluated and items with values > 2 were considered for removal. Items with outfit < 0.5 were less concerning [38]. Items were removed iteratively starting with those with the highest mean-square outfit misfit with standardized outfit statistics > 2. Fit statistics were recalculated and reevaluated until the items fit the model. Items with negative point-measure correlations were removed as these correlations indicated that the items were negatively polarized with the rest of the scale [33, 39]. In addition to item misfit, person misfit was also assessed. Similar to procedures in other studies [40] and proposed by Linacre [37], tables of most misfitting responses were examined, and for each analyses, 1 round of the most misfitting responses were removed and compared with the original model. If removing the misfitting responses did not improve model fit, the original dataset was retained. However, when model fit was improved, final analyses were conducted on the dataset with the misfitting responses removed. Consistent with other studies using Rasch, final decisions to retain or delete items were based on both statistical findings and theoretical reasoning for the items [41]. Unidimensionality, local independence, and monotonicity were examined at each iteration. See Table 1 for item difficulties and fit statistics for the final assessments.

**Table 1:** Rasch item difficulties and fit statistics

	Individual					Composite				
Item	Difficulty	SE	Outfit MNSQ	Outfit ZSTD	PMC	Difficulty	SE	Outfit MNSQ	Outfit ZSTD	PMC
**Functional HL**										
FHLD7	0.98	0.14	1.14	1.6	0.59	1.43	0.13	1.21	2.4	0.44
FHLD5	0.73	0.14	0.94	−0.7	0.63	1.21	0.13	1.00	0.1	0.50
FHLD3	0.62	0.14	1.12	1.5	0.57	1.12	0.13	1.42	4.2	0.37
FHLD6	−0.12	0.15	0.81	−2.0	0.62	0.47	0.14	0.92	−0.6	0.47
FHLD4	−0.94	0.17	0.82	−1.1	0.57	−0.40	0.17	0.72	−1.5	0.44
FHLD2	− 1.28	0.19	1.26	1.3	0.43	−0.92	0.20	0.68	−1.3	0.39
**Interactive HL**										
ICHLD13	2.91	0.16	1.31	1.6	0.62	1.99	0.13	1.15	1.7	0.45
ICHLD18	2.02	0.15	0.98	−0.1	0.66	1.36	0.13	1.01	0.1	0.50
ICHLD14	0.64	0.16	1.03	−0.2	0.59	0.23	0.15	0.75	−1.9	0.50
ICHLD17	0.35	0.17	0.93	−0.4	0.58	−0.02	0.16	0.78	−1.4	0.51
ICHLD8	−0.02	0.18	0.98	−0.1	0.53	−0.32	0.17	0.80	−1.0	0.43
ICHLD16	−0.26	0.19	0.87	−0.6	0.48	−0.53	0.18	0.85	−0.6	0.36
ICHLD6	−0.50	0.20	1.06	0.3	0.42	−0.92	0.20	0.81	−0.6	0.34
ICHLD9	−1.21	0.24	0.31	−2.6	0.53	−1.28	0.22	0.36	−2.5	0.45
ICHLD5	−1.87	0.29	0.32	−1.7	0.42	−1.92	0.28	0.25	−2.2	0.38
ICHLD15	−2.05	0.30	0.29	−1.7	0.42	−2.02	0.29	0.32	−1.7	0.33
**Critical HL**										
CRHLD6	0.81	0.14	1.16	1.9	0.52	0.81	0.14	0.98	−0.2	0.49
CRHLD2	0.47	0.15	1.03	0.4	0.51	0.50	0.14	0.99	0.0	0.47
CRHLPC11	0.36	0.10	1.11	0.7	0.58	0.42	0.10	1.40	1.7	0.47
CRHLP4	−0.04	0.10	0.97	−0.3	0.68	0.06	0.09	1.09	1.0	0.58
CRHLP5	−0.05	0.09	0.91	−0.7	0.68	0.05	0.09	0.88	−0.8	0.62
CRHLP7	−0.65	0.13	0.84	−1.1	0.59	−0.57	0.13	0.71	−1.7	0.54
ICCHLP3R	−0.89	0.13	0.85	−0.9	0.54	−0.80	0.13	0.75	−1.2	0.51

*HL* health literacy, *MNSQ* Mean-square, *PMC* point-measure correlation, *SE* standard error; *ZSTD* standardized statistic

Item-separation reliability statistics were examined to determine if the item difficulty range was sufficient with scores closer to 1 indicating good item separation. Wright [42] argued that the reliability of a test for a sample assumes symmetric ability which is rarely the case in health-related research and proposed an alternative method of calculating reliability, Wright's sample-independent reliability statistics, to be employed once measure calibration was completed [42] in place of Winsteps’ person reliability statistics. The procedures involved determining the number of strata across the scores then using this to calculate the sample-independent reliability: number of levels^2^ /1 + number of levels^2^. Number of strata and cutoff scores were determined by inflating the standard errors of the raw score by 10% then using twice the joint standard errors and the logit of the lowest and current raw scores to separate the scores into levels of performances. Sample-independent reliability was more appropriate for this study given that the sample was skewed in ability. As an exploratory step, reliability, strata and cutoff scores were calculated by age group (12–15 vs 16–18 years). Lastly, invariance of items across subpopulations were assessed by calculating uniform differential item functioning (DIF) for gender, age, and ethnicity. A minimum of 100 participants per group is required to detect DIF that is ≥0.5 logits and statistically significant [43]. Due to small sample sizes, age was grouped 12–15-years-old and 16–18-years-old to calculate DIF. For all DIF analyses, significance thresholds were set to *p* < 0.01 to account for multiple tests. The sample size for the Rasch model calculations was appropriate as models can be estimated with 99% confidence within 0.5 logits with a minimum sample of 108 to 243 [44]. For the Rasch Partial Credit Model, each response category surpassed the minimum requirement of 10 responses [45].

After finalizing the assessments, descriptive statistics were calculated. Adolescents 12–14 years were combined due to small n. Similarly, adolescents who identified as Asian, American Indian or Alaska Native/Native Hawaiian or Other Pacific Islander were combined due to small n. Convergent validity (whether two measures of constructs that should be related are related [46]) of the AAHL with an existing measure of FHL (NVS) was assessed by examining the correlations between the summed scores of both measures. Given that the NVS only measures FHL and the developed assessments measure IHL and CHL the relationships will be considered hetero-trait and therefore only moderate correlations were expected. Further, given the reason for developing a new FHL assessment for adolescents was that the NVS was originally developed for adults and relied on familiarity with nutrition labels, a moderate correlation was also expected between the existing and developed measures of FHL. Criterion validity (whether the score on one measure is related to a direct outcome of the phenomenon [47]) was assessed by modeling logistic regressions predicting HL-related behaviors from AAHL after controlling for demographics. Effect sizes were also estimated by estimating receiver operating characteristic curves and transforming the area under the curves to *Cohen’s d* using tables proposed by Salgado [48]. Exploratory analyses to determine convergent and criterion validity by younger vs. older adolescents were explored. Note that given the small sample sizes, only the effect sizes were estimated (via the receiver operating characteristic curves) for criterion validity. To compare mean assessments scores and categorizations, independent t-tests and one-way ANOVAs and associated effect sizes were computed. Effect sizes were the mean differences between z-scores (e.g., IHL scores) across categories of the other assessments (e.g., FHL categories). Crosstabs and chi-squares were also computed to determine the relationship between categorizations across the assessments. In crosstabs where a cell was less than 5, Fisher’s exact tests were computed.

## Results

The sample consisted of 355 adolescents (Mean age = 16 years, SD = 1.34; 55% girls). All but one participant chose either male or female. The largest racial group was Other (~ 35%) and ~ 51% of the sample was non-Hispanic/Latinx. Only a subsample (*n* = 200) of participants completed the NVS as the scale was placed at different points of the measurement battery and adolescents who received the measurement battery (randomly) with the NVS placed last did not have sufficient time to complete it. Of those that completed the NVS, 35% had a high likelihood of limited literacy and 27% had adequate literacy. See Table 2 for descriptive statistics.

**Table 2:** Descriptives of the sample

Variable	n(%)	FHL M(SD)	F	IHL M(SD)	F	CHL M (SD)	F	Composite M (SD)	F
Gender			1.47		25.94***		6.30*		13.93***
Boys	136(45.2)	4.24(1.58)		7.54(2.12)		10.99(2.79)		22.84(5.53)	
Girls	165(54.8)	4.45(1.37)		8.61(1.43)		11.78(2.53)		25.03(4.25)	
Missing	54								
Age (years)			3.51**		1.46		2.60*		2.56*
12–14	57(18.7)	4.09(1.60)		7.77(2.15)		11.33(2.77)		23.22(5.58)	
15	50(16.4)	4.49(1.37)		8.33(1.64)		11.60(2.43)		24.47(4.48)	
16	63(20.7)	4.89(1.10)		8.44(1.64)		12.05(2.17)		25.36(3.85)	
17	101(33.1)	4.29(1.51)		8.13(1.84)		11.32(2.71)		23.99(5.03)	
18	34(11.1)	3.90(1.46)		7.72(2.07)		10.19(3.64)		22.23(6.09)	
Missing	50								
Age (categories)			0.01		0.03		0.33		0.06
Younger (12-15)	107(30.1)	4.28(1.50)		8.04(1.93)		11.45(2.61)		23.82(5.09)	
Older (16-18)	248(69.9)	4.29(1.51		8.08(1.86)		11.26(2.83)		23.97(5.02)	
Hispanic/Latinx			0.71		1.40		0.05		0.46
Yes	150(50.5)	4.30(1.49)		8.27(1.62)		11.50(2.63)		24.33(4.52)	
No	147(49.5)	4.44(1.44)		8.01(1.97)		11.43(2.66)		23.89(5.15)	
Missing	58								
Race			0.78		0.99		0.86		1.31
ANAANNHOPI	24(8.6)	4.13(1.46)		7.58(2.90)		10.79(3.71)		22.30(7.33)	
Black	61(21.9)	4.35(1.51)		7.95(1.96)		11.22(2.73)		23.52(5.18)	
White	66(23.7)	4.61(1.50)		8.14(1.77)		11.63(2.50)		24.52(5.08)	
Multiracial	31(11.1)	4.57(1.36)		8.43(1.71)		11.93(2.92)		24.96(5.02)	
Other^a^	97(34.8)	4.28(1.51)		8.28(1.52)		11.58(2.28)		24.39(4.05)	
Missing	76								
Newest Vital Sign			20.53***		23.71***		36.10***		41.54***
High likelihood limited literacy	70(35)	3.54(1.73)		7.03(2.15)		9.88(3.16)		20.51(4.15)	
Possibility of limited literacy	76(21.4)	4.61(1.15)		8.39(1.41)		12.08(1.85)		25.21(3.01)	
Adequate literacy	54(27)	5.12(1.20)		9.02(1.10)		13.28(1.09)		27.45(2.15)	
Missing	155								
Functional HL			737.69***		39.62***		55.53***		141.63***
Emerging	155(47.7)	2.97(1.09)		7.40(2.14)		10.20(3.15)		20.81(5.34)	
Expanding	170(52.3)	5.49(0.50)		8.68(1.40)		12.35(1.84)		26.54(2.80)	
Missing	30								
Interactive HL			77.89***		398.16***		170.95***		372.35***
Emerging	34(10.8)	2.53(1.50)		4.03(1.09)		6.88(2.37)		13.44(3.52)	
Expanding	281(89.2)	4.60(1.27)		8.56(1.27)		11.99(2.11)		25.26(3.34)	
Missing	40								
Critical HL			72.20***		168.71***		670.00***		448.52***
Emerging	56(17.5)	2.87(1.74)		5.57(2.08)		6.39(1.85)		15.10(4.19)	
Expanding	264(82.5)	4.61(1.28)		8.59(1.38)		12.37(1.50)		25.65(2.96)	
Missing	35								
AAHL Composite			78.04***		166.40***		225.44***		619.49***
Emerging	7(2.3)	1.71(1.11)		3.00(1.15)		4.00(2.16)		8.71(1.38)	
Expanding	44(14.7)	2.70(1.39)		5.43(1.69)		7.36(1.73)		15.50(2.57)	
Lower Bridging	175(58.3)	4.42(1.18)		8.33(1.19)		11.87(1.63)		24.61(2.01)	
Upper Bridging	74(24.7)	5.57(0.53)		9.64(0.56)		13.54(0.65)		28.74(0.79)	
Missing	55								

*ANAANNHOPI* Asian, Native American/Alaskan Native, Native Hawaiian/Other Pacific Islander, *AAHL* assessments of adolescent health literacy, *CHL* critical health literacy, *FHL* functional health literacy, *HL* health literacy, *IHL* interactive health literacy, *M* mean, *SD* standard deviation

^a^ 70 adolescents who identified as Other indicated they were Hispanic/Latinx; *p < 0.05; **p < 0.01; ***p < 0.001

### FHL

The revised FHL item bank contained 12 items assessing numeracy and reading skills mainly using a cafeteria menu and an over-the-counter prescription label. Six items were removed due to outfit misfit. Removal of the most misfitting person responses did not improve model fit, therefore all responses were retained. The final 6-item FHL assessment evaluated reading comprehension, reading charts, and numeracy skills (see Additional file 1). Point-measure correlations for the final assessment were 0.43–0.63 suggesting high correlations with person abilities. Assumptions of unidimensionality (eigenvalue = 1.4), local independence (correlations ≤ − 0.31), and monotonicity were met. No DIF was detected for gender, age, and ethnicity. Item separation reliability (0.97) was acceptable. The Wright sample-independent reliability statistic was 0.80 and the scores differentiated two distinct levels of performances: Emerging (scores 0–4) and Expanding FHL (scores 5–6). Wright sample-independent reliability and cutoff scores did not differ by age group (12–15 vs. 16–18 years). The Kuder-Richardson Formula 20 (KR-20) alpha was 0.63 which was below the 0.7 threshold for acceptability.

FHL scores (Mean = 4.29, SD = 1.51) differed significantly by age and NVS category. Specifically, adolescents 16-years-old had significantly higher scores than adolescents 12–14-years-old (Mean difference = 0.80, *p* = 0.030) and 18-year-olds (Mean difference = 0.98, *p* = 0.019). For NVS, adolescents with high likelihood of limited literacy had significantly lower FHL scores than adolescents with possibility of limited literacy (Mean difference = − 1.08, *p* < 0.001) and adequate literacy (Mean difference = − 1.58, *p* < 0.001). The assessment had convergent validity with the NVS in the general (*r* = 0.44, *p* < 0.001) sample and in the younger (*r* = 0.31, *p* = 0.013) and older (*r* = 0.51, *p* < 0.001) adolescent subsamples. Regarding criterion validity, the assessment was positively related to adolescents questioning truthfulness of health information found online (odds ratio [OR] = 1.31, 95% confidence interval [CI]:1.10,1.58, *d* = 0.43) and reading instructions before taking medicine (OR = 1.31, CI:1.02,1.69, *d* = 0.49). In the younger adolescent subsample adolescents, the *Cohen d* effect sizes estimated via the receiver operating characteristic curves for criterion variables and FHL were 0.34–0.39 indicating a small effect size while in the older adolescent sample, effect sizes were small to medium (0.44–0.57).

### IHL

The revised IHL item bank contained 15 items and 10 items were retained for the final assessment (see Additional file 2). The final assessment evaluated adolescents’ skills for interacting with providers, multiple sources of contradictory information, and using knowledge to inform current behavior. Four items were removed due to high outfit statistics and one item was removed due to low point-measure correlation. Removal of the most misfitting person responses improved model fit, therefore final model estimation was done on the dataset with misfitting responses removed. Point-measure correlations for the final assessment were 0.42–0.66. Assumptions of unidimensionality (eigenvalue = 1.6), local independence (correlations ≤0.30), and monotonicity were met. No DIF was detected for gender, age, and ethnicity. Item separation reliability (0.98) was acceptable. The Wright sample-independent reliability statistic was 0.80 and the scores differentiated two distinct levels of performances: Emerging (scores 0–5) and Expanding IHL (scores 6–10). Wright sample-independent reliability and cutoff scores did not differ by age group (12–15 vs. 16–18 years). The KR-20 alpha was 0.74.

IHL scores (Mean = 8.07, SD = 1.88) differed by gender and NVS category. Adolescent girls had significantly higher IHL scores than adolescent boys (Mean difference = 1.07, *p* < 0.001). Adolescents with high likelihood of limited literacy had significantly lower IHL scores than adolescents with possibility of limited literacy (Mean difference = − 1.36, *p* < 0.001) and adequate literacy (Mean difference = − 1.99, *p* < 0.001). Convergent validity with the NVS was established in the general (*r* = 0.43, *p* < 0.001) sample and in the younger (*r* = 0.36, *p* = 0.004) and older (*r* = 0.47, *p* < 0.001) adolescent subsamples. Regarding criterion validity, IHL was positively related to questioning the truthfulness of health information found online (OR = 1.43, CI:1.21,1.68, *d* = 0.67) and reading instructions before taking medicine (OR = 1.43, CI:1.16,1.77, *d* = 0.66). The *Cohen d* effect sizes for criterion variables and IHL were 0.75–0.84 in the younger sample and 0.55–0.62 in the older sample.

### CHL

The revised CHL item bank contained 9 items that assessed skills for engaging in advocacy around health issues and understanding how socioeconomic barriers may impact health decisions. Seven of the 9 items were retained for the final assessment (see Additional file 3). This assessment was evaluated using the Rasch Partial Credit Model. The response options were ranked from not at all CHL to collective advocacy (where applicable) skills, except for items CRHLD2 and CRHLD6 which were scored as incorrect or correct. One item was removed due to high misfit outfit mean-square statistics and a second item was removed due to extremely low point-measure correlation. Removal of the most misfitting person responses did not improve model fit, therefore all responses were retained. Point-measure correlations for the final assessment were 0.51–0.68. Assumptions of unidimensionality (eigenvalue = 1.4), local independence (correlations≤0.31), and monotonicity were met. No DIF was detected for gender, age, and ethnicity. Item separation reliability (0.95) was acceptable. The Wright sample-independent reliability statistic was 0.80 with the scores differentiating two distinct levels of performances: Emerging (scores 0–8) and Expanding (scores 9–14) CHL. Wright sample-independent reliability and cutoff scores did not differ by age group (12–15 vs. 16–18 years). The KR-20 alpha was 0.74. Note that the scores ranged from 0 to 14 though only 7 items were retained. This is because with Rasch Partial Credit Models each polytomous response option has a unique score that corresponds to degree of correctness.

CHL scores (Mean = 11.32, SD = 2.76) differed significantly by gender, age, and NVS category. Adolescent girls scored significantly higher than adolescent boys (Mean difference = 0.78, *p* = 0.013). Adolescents 16-years-old scored higher than 18-year-olds (Mean difference = 1.86, *p* = 0.017). Regarding the NVS, adolescents with a high likelihood of limited literacy scored significantly lower than adolescents with possibility of limited literacy (Mean difference = − 2.20, *p* < 0.001) and adequate literacy (Mean difference = − 3.40, *p* < 0.001) while adolescents with possibility of limited literacy scored lower than adolescents with adequate literacy (Mean difference = − 1.20, *p* = 0.011). NVS was significantly positively correlated with the CHL assessment (*r* = 0.52, *p* < .001), therefore convergent validity was established. Convergent validity was also established in the younger (*r* = 0.54, *p* < 0.001) and older (*r* = 0.52, *p* < 0.001) adolescent subsamples. Regarding criterion validity, CHL was positively related to questioning the truthfulness of health information found online (OR = 1.25, CI:1.12,1.40, *d* = 0.61) and reading instructions before taking medicine (OR = 1.27, CI:1.10,1.46, *d* = 0.66). The *Cohen d* effect sizes for criterion variables and CHL were 0.48–0.54 in the younger sample and 0.69–0.74 in the older sample.

### AAHL composite

A Rasch Partial Credit Model was estimated to evaluate how well the final items in the FHL, IHL, and CHL assessments fit in a single model. Items FHLD3 and FHLD7 had standardized outfit statistics above 2.0, however, outfit and infit mean-square fit statistics were in the acceptable range so no further action was required. Point-measure correlations were 0.33–0.62. Assumptions of unidimensionality (eigenvalue = 1.8), local independence (correlations≤0.42), and monotonicity were met. No DIF was detected for gender, age, and ethnicity. Item separation reliability (0.97) was acceptable. Wright sample-independent reliability statistic was 0.94 with the scores differentiating four levels of performances: Emerging (0–10), Expanding [11–19], Lower Bridging [20–27], and Upper Bridging [28–30]. Wright sample-independent reliability did not differ but cutoff scores were slightly different for the 12–15-year-old age group such that the Lower Bridging range was 20–26 and Upper Bridging was 27–30. The KR-20 alpha was 0.91. Similar to CHL, the score range was larger than the number of items as a Rasch Partial Credit Model was estimated and each polytomous response option had a unique score that corresponded to degree of correctness.

AAHL Composite scores (Mean = 23.92, SD = 5.04) differed significantly by gender, age, and NVS category. Adolescent girls scored significantly higher than adolescent boys (Mean difference = 2.19, *p* < 0.001). Adolescents 16-years-old had higher scores than 18-year-olds (Mean difference = 3.13, *p* = 0.044). Regarding the NVS, adolescents with a high likelihood of limited literacy scored significantly lower than adolescents with possibility of limited literacy (Mean difference = − 4.71, *p* < 0.001) and adequate literacy (Mean difference = − 6.94, *p* < 0.001) while adolescents with possibility of limited literacy scored lower than adolescents with adequate literacy (Mean difference = − 2.24, *p* = 0.013). The NVS was positively correlated with the AAHL Composite (*r* = 0.56, *p* < 0.001) in the general sample and in the younger (*r* = 0.52, *p* < 0.001) and older (*r* = 0.58, *p* < 0.001) adolescent subsamples establishing convergent validity. Regarding criterion validity, AAHL Composite scores were positively related to questioning truthfulness of health information found online (OR = 1.15, CI:1.08,1.22, *d* = 0.73) and reading instructions before taking medicine (OR = 1.15, CI = 1.06,1.24, *d* = 0.79). The *Cohen d* effect sizes for criterion variables and AAHL Composite were 0.73–0.78 in the younger sample and 0.69–0.78 in the older sample.

### Comparing FHL, IHL, CHL, and AAHL composite scores

Adolescents categorized as having Expanding FHL had higher IHL (*d* = 0.72), CHL (*d* = 0.84*)*, and composite HL (*d =* 1.38) than those categorized as Emerging. Adolescents categorized as having Expanding IHL had higher FHL (*d* = 1.61), CHL (*d* = 2.38), and composite HL (*d* = 3.51) than those categorized as Emerging. Adolescents categorized as having Expanding CHL had higher FHL (*d* = 1.27), IHL (*d* = 1.99), and composite HL (*d* = 3.31) than those categorized as Emerging. Adolescents categorized as having Upper Bridging composite HL had higher FHL scores than those categorized as Emerging (*d* = 6.52), Expanding (*d* = 3.03), and Lower Bridging (*d* = 1.12). Adolescents categorized as having Upper Bridging composite HL had higher IHL scores than those categorized as Emerging (*d* = 10.57), Expanding (*d* = 3.75), and Lower Bridging (*d* = 1.25). Adolescents categorized as having Upper Bridging composite HL had higher CHL scores than those categorized as Emerging (*d* = 11.10), Expanding (*d* = 5.28), and Lower Bridging (*d* = 1.19). Table 3 shows the crosstabs based on HL categorization and all chi-squares and Fisher’s exact tests were significant at the *p* < 0.001 level suggesting that there is a relationship between the assessments when categories are used.

**Table 3:** Cross tabs of categorizations of the sample based on health literacy cutoff scores

	Functional		Interactive		Critical		AAHL Composite			
	Emerging	Expanding	Emerging	Expanding	Emerging	Expanding	Emerging	Expanding	Lower Bridging	Upper Bridging
	**n (%)**	**n(%)**	**n(%)**	**n(%)**	**n(%)**	**n(%)**	**n(%)**	**n(%)**	**n(%)**	**n(%)**
Functional HL										
Emerging	–	–	31 (91.2)	111 (40.7)	43 (79.6)	104 (40.2)	7 (100)	40 (90.9)	89 (50.9)	1 (1.4)
Expanding	–	–	3 (8.8)	162 (59.3)	11 (20.4)	155 (59.8)	0 (0)	4 (9.1)	86 (49.1)	73 (98.6)
Interactive HL										
Emerging	31 (21.8)	3 (1.8)	–	–	27 (52.9)	7 (2.7)	7 (100)	25 (56.8)	2 (1.1)	0 (0)
Expanding	111 (78.2)	162 (98.2)	–	–	24 (47.1)	248 (97.3)	0 (0)	19 (43.2)	173 (98.9)	74 (100)
Critical HL										
Emerging	43 (29.3)	11 (6.6)	27 (79.4)	24 (8.8)	–	–	7 (100)	34 (77.3)	8 (4.6)	0 (0)
Expanding	104 (70.7)	155 (93.4)	7 (20.6)	248 (91.2)	–	–	0 (0)	10 (22.7	167 (95.4)	74 (100)
AAHL Composite										
Emerging	7 (5.1)	0 (0.0)	7 (20.6)	0 (0)	7 (14.3)	0 (0)	–	–	–	–
Expanding	40 (29.2)	4 (2.5)	25 (73.5)	19 (7.1)	34 (69.4)	10 (4)	–	–	–	–
Lower Bridging	89 (65)	86 (52.8)	2 (5.9)	173 (65)	8 (16.3)	167 (66.5)	–	–	–	–
Upper Bridging	1 (0.7)	73 (44.8)	0 (0.0)	74 (27.8)	0(0)	74 (29.5)	–	–	–	–

*AAHL* assessment of adolescent health literacy, *HL* health literacy; Percentages are percent within column for each type of health literacy; All chi-squares and Fisher’s exact tests were significant at *p* < 0.001

## Discussion

This study aimed to develop and validate test-based assessments of adolescents’ FHL, IHL, and CHL. Face and content validity were established using focus groups, expert review, and cognitive interviews in the early phases of the study. Construct validity was established using Rasch models. The final assessments fit their respective Rasch models and met the key Rasch assumptions of unidimensionality, local independence, and monotonicity. Key Rasch assumptions were also met when all items across the three assessments were entered into a single Rasch model. Each assessment had good convergent and criterion validity.

The FHL, IHL, and CHL assessments are measures of different aspects of HL. However, they are a related set of skills and Nutbeam [9] proposed that the order of the complexity and difficulty starts with FHL then IHL then CHL. Across the categories for each assessment, the scores of the alternative assessments were linear such that the highest category had the highest scores on the other assessments. Further, crosstabs of the assessment categories indicated a relationship between the assessments. These results support the relatedness of the different types of HL. Regarding the nested structure, we would expect that the largest cells in the crosstabs would be where there is congruence in categorization (e.g., Emerging FHL and Emerging CHL). This pattern was noted for the IHL, CHL, and AAHL Composite (Emerging/Expanding vs. Lower/Upper Bridging) categorization columns in Table 3. However, for the FHL categorization columns, more adolescents with Emerging FHL were categorized as Expanding IHL or CHL than Emerging IHL or CHL. A similar pattern was noted for AAHL Composite with more adolescents with Emerging FHL categorized as Lower Bridging AAHL Composite than Emerging or Expanding AAHL Composite.

In our item set, the difficulty level on the FHL items were relatively higher than those on the IHL and CHL. This is not surprising as measures of FHL assess numerical and reading skills which are highly academic in content while the other measures assess social, interpersonal, and “know how” that one can acquire via opportunities for modeling, scaffolding, and practice. This aligns with the argument that HL is a type of cultural health capital [49]. When our FHL assessment and the NVS were included in a single Rasch model, 5 of the 6 NVS items were more difficult than the highest difficulty item on our FHL assessment. Therefore, the FHL assessment performing differently than what is proposed theoretically is less likely to be due to our assessment’s difficulty. We propose that there are qualitative differences in how HL skills may be acquired and these differences may explain why FHL may be a more difficult skillset than IHL and CHL during adolescence. Test-based measures of IHL and CHL for adults to determine if the same patterns of difficulty are found in adults are needed.

Relatedly, we chose a cafeteria menu and an over-the-counter medication label rather than a nutrition facts label for the initial FHL assessment as we expected these would be more familiar to adolescents. In our experience using the NVS with younger adolescents (i.e., 13–14-year-olds), being presented with the nutrition label is overwhelming and anxiety-provoking for adolescents likely due to unfamiliarity with reading nutrition labels. Given that HL skills develop through experience [8] and that adolescents may have more experience reading a cafeteria menu than a nutrition label, we expected the familiarity of the menu would be more conducive to adolescents excelling at demonstrating their skills. This may also explain why adolescents performed better on the FHL measure when compared to the NVS and provides support for the need for HL measures developed specifically for adolescents rather than validating adult measures in adolescent samples. In cognitive interviews, the cafeteria menu tested slightly better than the over-the-counter medication label. However, responses on the over-the-counter medication label were inconsistent when evaluated in the Rasch model. We propose that exposure to cafeteria menus and over-the-counter medication labels differ with fewer adolescents having sufficient exposure to medication labels (compared to cafeteria menus) to not be overwhelmed when their reading and numeracy skills are tested using these labels.

Of Nutbeam’s [9] three HL concepts, CHL is the most complicated to operationalize. Sykes and colleagues [50] conducted a study on the conceptualization of CHL across discipline, time, place, and multiple types of users and found that definitions ranged from emphasizing higher order cognitive skills to empowerment and critical engagement to affect sociopolitical change. We attempted to represent the range of definitions from individual cognitive skills to collective advocacy to affect community health in our response options. Hence, the use of Rasch Partial Credit Model for the CHL assessment and the ranking of the options from not at all HL to collective advocacy (where applicable). This made for a more accurate assessment of the skill than would be estimated if the responses were dichotomized into correct and incorrect. Further, use of focus groups to elicit community health topics important to adolescents and modes of advocacy in which adolescents engaged or wish to engage allowed for a robust CHL assessment that was relatable and relevant to adolescents’ lived experiences.

As a secondary exploratory step, Rasch models were estimated separately for younger (12–15 years) and older (16–18 years) adolescents for the purposes of exploring if the cutoff scores were similar in both groups. For FHL, IHL, and CHL assessments, the cutoff scores were similar. However, for the AAHL Composite, the cutoff score between Lower and Upper Bridging was lower for the younger group. Given that the sample for the younger age group was smaller than what is required for estimating the Rasch model [44], we propose that these findings be replicated in an adequate sample before making assumptions for this discrepancy. We, however, hypothesize that with a sufficient sample size to study each age separately, it is likely that the cutoff score will vary with higher cutoff scores for older age given that HL is developmental [8].

The effect sizes for predicting HL behaviors from FHL, IHL, and CHL in the general sample ranged from 0.43–0.67 indicating small to medium effect sizes. Small to large effect sizes were estimated in the younger adolescent subsample (0.34–0.84) and small to medium effect sizes in the older adolescent subsample (0.44–0.74). These effect sizes suggest that the final objective assessments have utility in predicting behavior and for assessing HL skills necessary for engaging in applied HL. The Composite score was a relatively stronger indicator of the HL behaviors (general sample = 0.73–0.79, younger subsample = 0.73–0.78, and older subsample = 0.69–0.78) suggesting that assessing these three types of HL together is a better indicator of HL skills than FHL alone as is common in both the adult and adolescent literature [2, 5, 11]. Our analyses reinforce Sørenson et al.’s [8] definition of HL as our assessments tested “do you have the skills?” and the relationship between these skills and applied use was confirmed through acceptable effect sizes.

Noteworthy are the significant differences in scores by demographic characteristics. For age, adolescents 16-years-old scored higher than 12–14-year-olds and 18-year-olds on the FHL assessment, and higher than 18-year-olds on the CHL, and AAHL Composite assessments. Given that the data was collected in a high school setting, there is a possibility that some 18-year-olds may still be enrolled in high school due to lower achievement which might be a product of intelligence and environment [51]. An environment not conducive to achievement (e.g., lower parental income, lower parental education [51, 52]) may also be less conducive to developing HL skills due to fewer opportunities for developing cultural health capital [49]. Regarding gender, girls scored higher than boys on IHL, CHL, and AAHL composite assessments. Interestingly, studies to date are contradictory on gender differences in HL, and most of this research focus on FHL [53–55]. Our findings reiterate the importance of measuring aspects of HL beyond FHL and suggest that adolescent boys and girls may require different HL interventions.

To date, test-based HL measures that have been validated in adolescents were all initially developed for adult populations. A major strength of this study is that the adolescent HL assessments were designed with consideration and inclusion of adolescents’ lived experiences. This study also provides the first test-based measures for IHL and CHL as well as core qualities of HL (AAHL Composite) as described by Sørenson and colleagues [8] across the adolescent and adult HL literature. Though these measures were developed for and in collaboration with adolescents, the items in the measures are relevant to both adolescent and adult populations. Therefore, future studies should assess the validity of these assessments with adult samples especially given the lack of test-based measures of the core qualities of HL in the literature.

This study is not without limitations. First, the sample ability was skewed due to adolescents being enrolled in health classes with some having interest in health careers. However, the sample-independent reliability corrected for this and allowed for identification of multiple strata of the constructs. Relatedly, the KR-20 for FHL was below the acceptable range of 0.70 despite sample-independent reliability being in acceptable range. KR-20 is influenced by test length and difficulty with shorter tests or tests skewed in difficulty having lower KR-20, respectively. Future studies should replicate the validity of the assessments in a sample of adolescents with more diverse person ability. Second, these assessments were developed using a convenience sample, therefore generalizability is limited. Note, however, that because Rasch analysis is sample-independent, this limitation is less concerning than if classical test theory was used. Third, though the sample size was sufficient for conducting the Rasch analysis, it was insufficient for calculating DIF for each age and for race. Despite this, the racial diversity of participants throughout all phases of the measurement development is a unique strength of the study. Future studies should be amply powered to explore item invariance for race and other demographic variables of interest such as parent education and family household income. These assessments should also be employed in longitudinal designs to determine their predictive validity and ability to detect change.

These FHL, IHL, and CHL assessments and their composite have utility in multiple settings. In school settings, health teachers may use students’ scores and categorization to identify curricular needs as well as to assess proficiency before and after relevant coursework. In medical settings, the assessments may be used to identify adolescents who may need additional support navigating their health care especially in cases where adolescents have the option to see providers without parental consent/attendance. This is particularly important as most states have minor consent laws for sexual health (adolescents may see providers about sexual-health related issues without parental consent) and some states have minor consent laws beyond sexual health [56, 57]. Interventionists may also use the assessments to identify HL intervention needs for adolescents as well as to ensure that their non-HL interventions are effective across the range of HL skills.

## Conclusion

This study aimed to develop test-based measures of adolescents’ FHL, IHL, and CHL. The finalized assessments met all the assumptions of Rasch, and had good model fit, and convergent and criterion validity. These assessments are the first test-based measures of adolescents’ IHL and CHL, the first test-based measure of FHL designed specifically for adolescents, and first composite test-based assessment of the core qualities of HL. These assessments have utility in multiple settings and will contribute significantly to how these constructs are studied and intervened on in future studies and programs.

## Supplementary Information

**Additional file 1.**

**Additional file 2.**

**Additional file 3.**

**Additional file 4.**

## Data Availability

The dataset analysed during the current study are not publicly available given that it is a relatively small dataset of a protected population but are available from the corresponding author on reasonable request.

## Abbreviations

AAHL: Assessments of adolescent health literacy
CHL: Critical health literacy
CI: Confidence interval
DIF: Differential item function
FHL: Functional health literacy
HL: Health literacy
IHL: Interactive health literacy
KR-20: Kuder-Richardson Formula 20
NVS: Newest vital sign
OR: Odds ratio
