# Supplementary material for: Development and validation of assessments of adolescent health literacy: a Rasch measurement model approach
Source: BMC Public Health. 2022 Mar 25;22:585. doi: 10.1186/s12889-022-12924-4 (PMC8953064; doi:10.1186/s12889-022-12924-4)
Supplement: Supplementary file 1 — Additional file 1. [file 12889_2022_12924_MOESM1_ESM.docx]

Additional file 1. Final Functional Health Literacy Assessment

The correct answers are italicized and scored 1. Incorrect responses are scored 0.

| # | VARIABLE NAME | QUESTION | SCORES: ANSWER CHOICES |
| --- | --- | --- | --- |
| 1 | FHLD2  Numerical calculation | 5ml=1 teaspoon  10ml = ____ | 0: 1 tablespoon  0: 1 teaspoon  *1: 2 teaspoons*  0: 2 tablespoons |
| 2 | FHLD3  Reading comprehension | An unhealthy blood pressure reading for an adult is above 120/80. Which of these is an unhealthy blood pressure reading? | 0: 115/70  0: 120/78  0: 96/64  *1: 150/97* |
|  | *Use the table and legend below to answer the next four questions (FHLD4-FHLD7).*  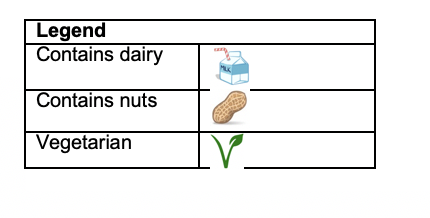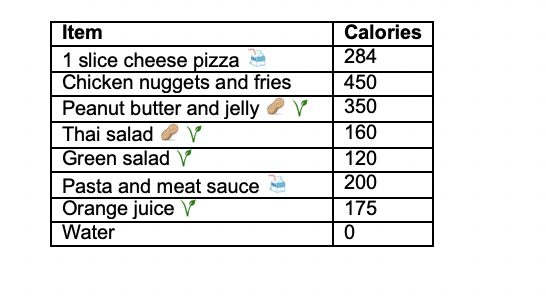 | | |
| 3 | FHLD4  Reading charts | *Use the table and legend below to answer the next five questions.*  Chelsea is allergic to dairy and nuts. Which of the following meals can Chelsea eat? | 0: Thai salad  0: Pasta and meat sauce  *1: Chicken nuggets and fries*  0: Cheese pizza |
| 4 | FHLD5  Reading comprehension, Numerical calculation | Sean can only eat up to 500 calories at lunch time. Which of the following contains too many calories to be Sean’s lunch? | 0: Cheese pizza and orange juice  *1: Peanut butter and jelly and orange juice*  0: Chicken nuggets and fries, and water  0: Pasta and meat sauce, green salad, and orange juice |
| 5 | FHLD6  Reading charts, numerical calculation | Jared is allergic to dairy and nuts. He can eat up to 200 calories at lunch. Which of the following meals can he eat for lunch? | 0: ½ serving chicken nuggets and fries  0: Pasta and meat sauce  0: Thai salad  *1: Green salad* |
| 6 | FHLD7  Numerical calculation | Maria got ½ peanut butter and jelly sandwich, a green salad, and an orange juice for lunch. How many calories are in her lunch? | 0: 605  *1: 470*  0: 525  0: 450 |
