# Supplementary material for: Development and validation of assessments of adolescent health literacy: a Rasch measurement model approach
Source: BMC Public Health. 2022 Mar 25;22:585. doi: 10.1186/s12889-022-12924-4 (PMC8953064; doi:10.1186/s12889-022-12924-4)
Supplement: Supplementary file 2 — Additional file 2. [file 12889_2022_12924_MOESM2_ESM.docx]

Additional file 2. Final Interactive Health Literacy Assessment

The correct answers are italicized and scored 1. Incorrect responses are scored 0.

|  | VARIABLE NAME | QUESTION | SCORES: ANSWER CHOICES |
| --- | --- | --- | --- |
| 1 | ICHLD5  Interaction with provider, interaction with multiple sources of contradictory information | Every time Kailey tests her blood pressure at home it is in the high range. Every time the nurse tests her blood pressure in the clinic, it is in the normal range. Should Kailey tell the nurse about her blood pressure readings at home? | *1: Yes*  0: No |
| 2 | ICHLD6  Interaction with provider, interaction with multiple sources of contradictory information | Every time Jordan tests their blood pressure at home it is in the normal range. However, every time they go to the doctor their blood pressure reading is in the high range.  Should Jordan ignore their blood pressure readings at home? | 0: Yes  *1: No* |
|  |  | *The two next questions are based on the following scenario:*  In the past Shane’s doctor advised her against taking diet drugs because of side effects. Today Shane saw a social media post for a diet drug with good results. |  |
| 3 | ICHLD8  Use of prior knowledge/communication to inform current behavior | The post says there are no side effects. Should Shane take the diet drug? | 0: Yes  *1: No* |
| 4 | ICHLD9  Provider communication | If Shane plans to take the diet drug. Should she tell her doctor about her plan to take the diet drug? | *1: Yes*  0: No |
| 5 | ICHLD13  Contradictory information, multiple sources, questionable reliability of source | Over the past week Jay saw the following signs:  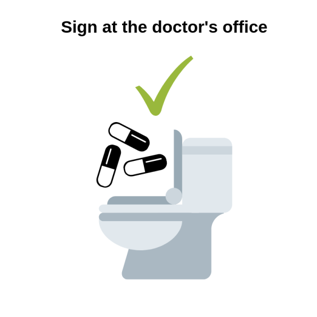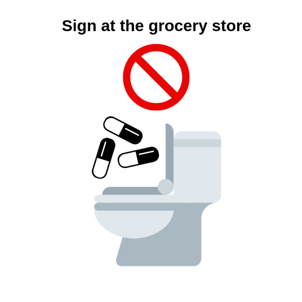  Jay has some pills she needs to get rid of. What should Jay do? | 0: Flush pills down the toilet.  0: Don’t flush pills down the toilet.  *1: Do more research on the issue before making the decision.*  0: Give the pills to someone who needs it. |
|  |  | *The next two questions are based on the following scenario:*  Eli went to the school nurse for his prescription medication to treat his migraine. The instructions from his doctor states take the medication every 4-6 hours. His migraine worsened 2 hours after taking the medication. |  |
| 6 | ICHLD14  Provider communication | What should Eli do? | 0: Check the internet to see if it is safe to take more medication.  *1: Tell the school nurse that the medication is not working*.  0: Ask the school nurse for more medication.  0: Take over-the-counter pain medication. |
| 7 | ICHLD15  Decision-making about interacting with provider | Eli has been taking over-the-counter pain medication in addition to his migraine medication. He has an upcoming doctor’s appointment but is feeling better. What should Eli do? | *1: Still go to the doctor*  0: Cancel his doctor’s appointment |
| 8 | ICHLD16  Use of prior knowledge/communication to inform current behavior | Mikey’s dentist gives him a mouthwash with specific instructions about not eating for 30 minutes after use. He changed dentists. His new dentist gave him a store brand version of the same mouthwash but no instructions. Should Mikey eat 15 minutes after using this mouthwash? | 0: Yes  *1: No* |
|  |  | *The next two questions are based on the following scenario:*  Kathy’s family usually eats dinner together. They eat lots of fried foods and few vegetables. Kathy wants to eat healthier – more fruits and vegetables, less fried foods. |  |
| 9 | ICHLD17  Communicating with family about health | How should Kathy interact with her family about wanting to eat healthier? | 0: Tell her family that they eat horribly.  0: Avoid foods she doesn’t want to eat.  *1: Tell her family she wants to change her eating behavior.* |
| 10 | ICHLD18  Communicating with family about health, use of knowledge to inform health | Kathy’s family wants to eat healthier too. How best can Kathy help her family eat healthier? | 0: Tell the family why she wants to change her eating habits.  0: Watch healthy cooking shows with her family.  *1: Suggest the family see a nutritionist.*  0: Suggest the family go on a low carbohydrates diet |
