# Supplementary material for: Development and validation of assessments of adolescent health literacy: a Rasch measurement model approach
Source: BMC Public Health. 2022 Mar 25;22:585. doi: 10.1186/s12889-022-12924-4 (PMC8953064; doi:10.1186/s12889-022-12924-4)
Supplement: Supplementary file 3 — Additional file 3. [file 12889_2022_12924_MOESM3_ESM.docx]

Additional file 3. Final Critical Health Literacy Assessment

| # | VARIABLE NAME | QUESTION | ANSWER CHOICES | SCORING |
| --- | --- | --- | --- | --- |
| 1 | CRHLD2 | Darryl lives in a neighborhood with no supermarkets. He usually shops for food at the gas station. Darryl wants to eat more fresh fruits and vegetables, but these are not sold at the gas station.  What can Darryl do to change the availability of fruits and vegetables in his community? | 1: Find the healthiest option at the gas station  2: Write a letter to the mayor about the problem | IF 1, Scored = 0.  IF 2, Scored = 1. |
| 2 | CRHLP4 | Jason read that sleep problems in teens are common. He thinks it is unfair that school starts at 7am for teens. Which of the following actions would be most successful in changing the school start time? | 1: Protest the school start time by showing up late to school every day.  2: Discuss his sleep problem with his school counselor.  3: Tell his parent about how unfair the school start time is.  4: Collect signatures from students to ask that school officials start school later. | IF 1, Scored = 0.  IF 2, Scored = 2. (engage with others with likely some authority)  IF 3, Scored = 1. (engage with others with likely little authority over the matter)  IF 4, Scored = 3. (collective advocacy) |
|  |  | *The next two questions are based on the following scenario:*  Laura lives in an apartment building and has a low income. Her neighborhood only has one public playground. She takes her children to this playground every afternoon after school. She just learned that the city council plans to turn the playground into a dog park. Laura believes that this is unfair. |  |  |
| 3 | CRHLP5 | What should Laura do? | 1: Take her children to a playground in another neighborhood.  2: Pay to enroll her children in an afterschool program.  3: Do nothing.  4: Protest the dog park at the next city council meeting | IF 1, Scored = 2. (more feasible than paying but income might still be barrier)  IF 2, Scored = 1. (ignores income restrictions)  IF 3, Scored = 0.  IF 4, Scored = 3. (advocacy for self and community) |
| 4 | CRHLD6 | Laura got neighbors to sign a letter to save the playground. Whom should she send the letter to? | 1: The mayor’s office  2: The dog park developers  3: The Department of Children Protection | IF 1, Scored = 1.  IF 2, Scored = 0.  IF 3, Scored = 0. |
| 5 | CRHLP7 | Mark has asthma. The mold in their apartment makes Mark’s asthma symptoms worse. They cannot afford to remove the mold or move to a new apartment.  What can Mark’s family do to help all the children in their apartment building with asthma? | 1: Nothing, only medications can help.  2: Apply for low-income housing so they can move.  3: Organize a group of parents to discuss the mold problem with the building owner. | IF 1, Scored = 0.  IF 2, Scored = 1. (helps self)  IF 3, Scored = 2. (collective advocacy to help all) |
|  |  | *The next two questions are based on the following scenario:*  A doctor recommends that a child exercise 60 minutes per day to lose weight. The doctor gives the family a list of outdoor exercise ideas. The child’s family lives in a low-income neighborhood with no safe parks and no other outside play areas. |  |  |
| 6 | ICCHLP3R | What should the family do now? | 1: Exercise in the unsafe park.  2: Move to another neighborhood with safe parks.  3: Search for free exercise programs/classes for kids. | IF 1, Scored = 0.  IF 2, Scored = 1. (not likely feasible)  IF 3, Scored = 2. (feasible, considerate of restrictions) |
| 7 | CRHLPC11 | The child currently gets most of their physical activity during recess. The family learns that the child’s school will no longer have recess. What should the family do? | 1: Switch to a different school.  2: Gather a group of parents to meet with the principal to discuss the issue.  3: Meet with the school principal alone and demand they keep recess.  4: Buy exercise equipment for the child to use at home. | IF 1, Scored = 0. (not likely possible)  IF 2, Scored = 2. (collective advocacy)  IF 3, Scored = 1. (self-advocacy less likely to bring about change)  IF 4, Scored = 0. (not likely possible) |
