# Supplementary material for: Development and validation of assessments of adolescent health literacy: a Rasch measurement model approach
Source: BMC Public Health. 2022 Mar 25;22:585. doi: 10.1186/s12889-022-12924-4 (PMC8953064; doi:10.1186/s12889-022-12924-4)
Supplement: Supplementary file 4 — Additional file 4. [file 12889_2022_12924_MOESM4_ESM.docx]

Steps for determining cutoff scores

1) Retrieve the Score table (Table 20.1 in Winsteps)

2) Inflate the standard errors of the raw scores in the table by 10%

3) Then apply the following equation starting with the lowest raw score (example 0):

Logit measure of lowest score + 2√(adjusted standard error of lowest score^2^ + adjusted standard error of current score^2^)

4) Compare the result of the equation to the logit measure for the current score:

a) If greater than logit measure for the current score, then score belongs to the same strata as previous score, that is the first strata. Repeat equation for the next raw score on the test only changing the adjusted standard error of current score. Compare the results to the logit measure for the current score and repeat procedures until the result of the equation is less than the logit measure for the corresponding current score. The last score in which the logit measure is larger than the result of the equation is the upper limit for the strata.

b) If less than logit measure for the current score, then score belongs to a new strata and is considered the lowest score in the strata. Repeat equation in step 3 for subsequent scores with the new lowest logit measure and adjusted standard error of lowest score corresponding to the lowest score in the strata. Follow process in 4a and 4b until all scores are assigned to a stratum.

Please see an illustration of these procedures at <https://www.rasch.org/rmt/rmt144k.htm>
